# Divergence within the Taxon ‘*Candidatus* Phytoplasma asteris’ Confirmed by Comparative Genome Analysis of Carrot Strains

**DOI:** 10.3390/microorganisms12051016

**Published:** 2024-05-17

**Authors:** Rafael Toth, Anna-Marie Ilic, Bruno Huettel, Bojan Duduk, Michael Kube

**Affiliations:** 1 Department of Integrative Infection Biology Crops-Livestock, University of Hohenheim, 70599 Stuttgart, Germany; rafael.toth@uni-hohenheim.de (R.T.); annamarie.ilic@uni-hohenheim.de (A.-M.I.); 2 Max Planck Genome Center, 50829 Cologne, Germany; huettel@mpipz.mpg.de; 3 Institute of Pesticides and Environmental Protection, 11080 Belgrade, Serbia; bojan.duduk@pesting.org.rs

**Keywords:** carrot, hybrid assembly, virulence factors, phylogeny

## Abstract

Phytoplasmas are linked to diseases in hundreds of economically important crops, including carrots. In carrots, phytoplasmosis is associated with leaf chlorosis and necrosis, coupled with inhibited root system development, ultimately leading to significant economic losses. During a field study conducted in Baden-Württemberg (Germany), two strains of the provisional taxon ‘*Candidatus* Phytoplasma asteris’ were identified within a carrot plot. For further analysis, strains M8 and M33 underwent shotgun sequencing, utilising single-molecule-real-time (SMRT) long-read sequencing and sequencing-by-synthesis (SBS) paired-end short-read sequencing techniques. Hybrid assemblies resulted in complete de novo assemblies of two genomes harboring circular chromosomes and two plasmids. Analyses, including average nucleotide identity and sequence comparisons of established marker genes, confirmed the phylogenetic divergence of ‘*Ca*. P. asteris’ and a different assignment of strains to the 16S rRNA subgroup I-A for M33 and I-B for M8. These groups exhibited unique features, encompassing virulence factors and genes, associated with the mobilome. In contrast, pan-genome analysis revealed a highly conserved gene set related to metabolism across these strains. This analysis of the Aster Yellows (AY) group reaffirms the perception of phytoplasmas as bacteria that have undergone extensive genome reduction during their co-evolution with the host and an increase of genome size by mobilome.

## 1. Introduction

Bacteria of the provisional taxon ‘*Candidatus* Phytoplasma’ are phloem-limited parasites [1] that infect a wide range of plant species, including many important crops as well as ornamentals and wild hosts. These parasites of the Mollicutes class are transmitted by phloem-sucking insect vectors in general [2,3], by vegetative propagation and in some rare cases by seed transmission [4,5]. Phytoplasmosis often manifests as leaf yellowing, hindered organogenesis, growth abnormalities, and general plant decline. As a consequence, this can lead to drastic yield losses in agriculture [6]. This is also true for phytoplasmosis of carrots (*Daucus carota* ssp. *sativus* L.), which has been reported in various cropping areas worldwide. Molecular screenings using PCR-based methods and phylogenetic analysis show that infections in European carrot production areas are mainly associated with ‘*Ca*. P. asteris’ strains that are also known as aster yellows (AY) phytoplasmas [7,8,9,10,11,12].

Infected carrot plants exhibit symptoms such as yellowing, reddening, necrosis of leaves, proliferation, and reduced taproot size. Such symptoms have been linked to the secretion of several phytoplasma effector proteins [13,14,15,16]. ‘*Ca*. P. asteris’- caused diseases have been reported in more than 300 species in 38 families of plants [3], and this taxon is the first and most extensively analysed phytoplasma species in terms of genome and pathogen–host interaction. Among the analysed genomes are ‘*Ca*. P. asteris’ strains infecting various hosts, including onion, lettuce [17,18], maize [19], grapevine (acc. no. CP035949), rapeseed [20], paulownia [21], and mulberry [22], but despite its importance, no carrot-associated strain has been characterised to date.

Herein, we provide new insights into the AY group by analysing the genomes of the two ‘*Ca*. P. asteris’ strains M8 and M33, were obtained during a field study in a carrot cultivation area in Baden-Württemberg, Germany. The complete genomes of the strains were reconstructed and compared with respect to their phylogenetic assignment and the common and distinct genetic repertoires of the previously described complete genomes of the AY group. Further, our results provide complex insights into the pathogen–host interaction and dependency of the phytoplasmas causing aster yellows disease.

## 2. Materials and Methods

### 2.1. Plant Material and DNA Extraction

**Figure 1:**
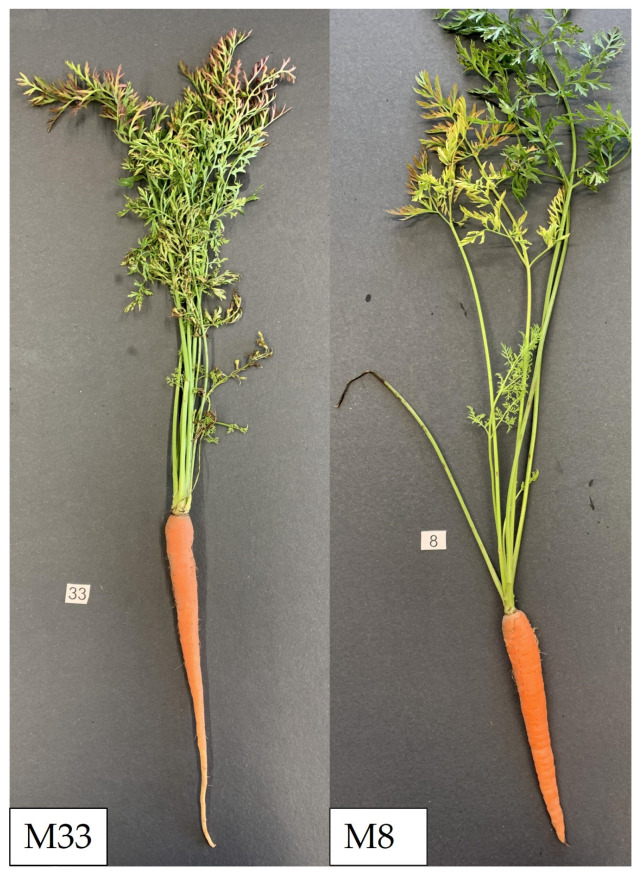
Carrots infected with ‘*Ca*. P. asteris’ strains M8 and M33 with leaves showing chlorosis, partial reddening, and terminal necrosis. In addition, infection with M33 shows a thin root (“rat tail” symptom).

Phytoplasmosis of carrots associated with ‘*Ca*. P. asteris’ strains are frequently ob-served in the south of Germany [23]. In August 2019, symptomatic carrots (*Daucus carota* subsp. *sativus*) were collected from a single carrot plot with 0.5% symptomatic plants in Langenau (Baden-Württemberg, Germany). Symptomatic carrots were collected and individually tested for ‘*Ca*. P. asteris’ by PCR and confirmed by sequencing the amplicons [24]. Two samples were selected for genome sequencing (Figure 1). Total DNA was purified using the cetyltrimethylammonium bromide (CTAB) plant DNA extraction protocol [25]. The DNA concentrations of the extracted DNA samples were quantified using a Qubit fluorometer (Thermo Fisher Scientific, Waltham, MA, USA). Infection was detected through endpoint PCR utilising the universal primers P1 and P7 for partial amplification of the phytoplasma rRNA operon [26,27].

### 2.2. Genome Sequencing

Shotgun sequencing of extracted DNA from infected carrot samples M8 and M33 (Figure 1) was performed separately for each sample using two sequencing technologies. To generate short reads with paired ends, Illumina sequencing [28] was carried out on the HiSeq 2500 platform (Illumina, San Diego, CA, USA). For long-read sequencing, single-molecule real-time sequencing (SMRT) sequencing [29] was performed on the Sequel IIe platform (Pacific Biosciences, Menlo Park, CA, USA). First, DNA was enriched for longer fragments through a 0.45% (*v*/*v*) PB AMPure bead purification step (Pacific Biosciences). Barcoded libraries were then prepared according to the manufacturer’s protocol “Preparing HiFi Libraries from Low DNA Input Using SMRTbell Express Template Prep Kit 2.0” (Pacific Biosciences). Libraries were equimolarly pooled and sequenced on a Sequel II device (Pacific Biosciences) using a Sequel II binding kit 2.0, Sequel II sequencing chemistry 2.0, and an 8M ZMW SMRT cell for 30 h (Pacific Biosciences). Sequencing data were demultiplexed and high-fidelity data were generated using the SMRTlink Suite v.9.0 (Pacific Biosciences) with default settings. The NGS approaches were conducted by the Max Planck Genome Centre Cologne (Cologne, Germany).

### 2.3. Hybrid Genome Assembly and Quality Assessment

The following analyses were also carried out individually for each strain. The short reads derived from each Illumina sequencing were mapped to the genome of ‘*Ca*. P. asteris’ strain RP166, which served as a reference to assign the reads obtained from the metagenomic DNA templates. Mapping and extraction of the reads were performed using the short-read to reference genome mapping tool as part of the CLC Genomic Workbench v. 22. (QIAGEN, Aarhus, Denmark) with default mapping parameters.

To reconstruct the chromosomes and plasmids of ‘*Ca*. P. asteris’ strains M8 and M33, the selection of mapped Illumina read pairs along with all generated SMRT reads were corrected, trimmed, and assembled to contigs using Canu assembler v. 1.9 [30]. The number of incorporated reads and the calculation of the sequencing coverage were taken from the Canu report files. Default correction and trimming parameters were used, while hybrid assembly was performed using the parameters ‘haplotype’ for Illumina reads and ‘pacbio-corrected’ for SMRT reads, setting an estimated genome size of 0.8 Mb. In order to identify phytoplasma replicons, all contigs were sorted by length using seqtk v. 1.3 (https://github.com/lh3/seqtk (accessed on 27 June 2022)) and split into two separate data sets for further evaluation. Contigs >5 kb kilobases were compared against a custom-made subset of protein databases containing all proteins assigned to *D*. *carota* subsp. sativus and to the phylum Mycoplasmatota using BLASTX [31]. Contigs <5 kb were compared with the UniProt Reference Cluster 100 (UniREF100) [32] using the DIAMOND high-throughput aligner v. 2.0.15 [33], applying the ‘fast’ parameter to increase the throughput of the larger dataset of contigs <5 kb. BLASTX and DIAMOND outputs were parsed using the Metagenome Analyzer (MEGAN) v. 6 [34] for taxonomic binning with default parameters enabling the identification of phytoplasma chromosomes and plasmids. Sequence coverage was extracted from the assembly output of Canu to assess the reliability and integrity of the assemblies [30]. Overlaps of circular constructs were confirmed using BLASTN [31], with default settings, and manually removed in the Artemis Genome Browser [35]. The chromosome start was set to the gene *dnaA* based on the cumulative G+C skew minimum, which was calculated in the Artemis Genome Browser [35]. For plasmids, the gene *rep* was set to position one.

Genome completeness was also estimated by comparative analysis of the protein content using the Benchmarking Universal Single-Copy Orthologs (BUSCO) v. 5.4.6 software [36]. The analysis was performed using the nucleotide sequences of the chromosomes of ‘*Ca*. P. asteris’ M8 and M33 along with all available complete chromosome sequences of ‘*Ca.* P. asteris’ and compared with a dataset of 151 conserved orthologues of the class Mollicutes on the Galaxy Europe server (https://usegalaxy.eu (accessed 12 August 2022)). In the absence of any further specifications, the default parameters were used.

### 2.4. Functional Annotation

The chromosomes and plasmids were functionally annotated using the Rapid Annotation using Subsystem Technology (RAST) v.2.0 pipeline [37]. Annotation parameters were set to translation table eleven and the NCBI taxonomy ID: 85,620 of ‘*Ca*. P. asteris’. Annotations were manually curated for missing, overlapping, or frameshifted ORFs. This analysis was conducted using BLASTP [31] comparison of the deduced protein sequence against the NRProt (from GenBank Release 251, www.ncbi.nlm.nih.gov (accessed on 19 August 2022)) and UniRef100 [32] databases. Adjustments were made in Artemis [35]. The deduced proteins were functionally characterised and used for metabolic reconstruction by applying BlastKOALA [38], the MetaCyc database v. 23.1 [39] and InterPro v. 86.0 [40]. Structural non-coding RNAs, such as tRNAs and rRNAs, were predicted using the software tRNAscan-SE v2.0.10 [41] and RNAmmer [42]. Membrane and secreted proteins were predicted with Phobius v. 1.01 [43] and InterPro [40]. Finally, the annotations of ‘*Ca*. P. asteris’ M8 and M33 were evaluated again using the Microbial Genome Submission check service from NCBI (https://www.ncbi.nlm.nih.gov/genomes/frameshifts/frameshifts.cgi (accessed on 6 June 2023)). Default parameters were used unless otherwise noted.

### 2.5. Phylogenetic and Functional Comparison

The M8 and M33 chromosomes were compared with all available complete chromosome sequences belonging to the taxon ‘*Ca*. P. asteris’, retrieved from NCBI (taxonomy ID: 85620) in terms of phylogeny and potential functions. For a phylogenetic analysis at the chromosome level, the average nucleotide identity (ANI) [44] was calculated based on a whole chromosome alignment and the neighbour-joining method [45] within the CLC Genomic Workbench v. 22 (QIAGEN Aarhus, Denmark) with default parameters. To verify the ANI results a whole chromosome sequence synteny analysis was conducted with the multiple genome aligner Mauve v. 20150226 [46] and the Artemis Comparison Tool (ACT) [35]. ACT analysis was calculated based on BLASTN [31] comparison M8-format outputs. Moreover, sequence identity analysis at the single gene level was performed with recently published marker genes (namely, 16S rRNA, *tufB*, *groEL*, *secY*, and *secA*) and the chromosomal region comprising the genes *rplV* to *rpsC*, according to current recommendations [47]. For each marker gene, an identity matrix based on multiple sequence alignments was calculated using BioEdit v. 7.2.5 [48] on default settings. The 16S rDNA phylogeny was analysed using Molecular Evolutionary Genetic Analysis (MEGA) v. 10.2 [49] with the maximum likelihood [50] and neighbour-joining method [45] based on a multiple sequence alignment generated in MEGA. Bootstrapping was performed with 1000 iterations. The threshold for a significant score to be included in the phylogenetic tree was set to 70%. OrthoFinder v. 2.5.5 [51] was used for pan-genome analysis, which included predicting orthologs, paralogs, and unique coding sequences (CDS) from assigned orthogroups that included all coding sequences (without pseudogenes) of the AY group members studied. Unassigned CDS were considered as unique CDS. Default parameters have been used unless otherwise stated.

## 3. Results

### 3.1. Sequencing and Hybrid Assembly

Illumina sequencing generated 2,116,564 paired-end short reads for strain M8 and 2,168,250 paired-end short reads for strain M33. A total of 223,496 reads for M8 were positively mapped on the genome of the strain RP166, whereas for M33 480,718 reads were selected for genome assembly input (Table 1). SMRT sequencing produced 191,988 long reads for strain M8 and 105,166 long reads for M33. For chromosome assembly, 10,116 reads for strain M8 and 11,776 reads for strain M33 were used. Final phytoplasma chromosome contigs showed a total length of 772,691 bp for M8 and 657,324 bp for M33. Sequencing coverage of the chromosome contigs was 116-fold for M8 and 176-fold for M33.

### 3.2. Quality Assessment

**Figure 2:**
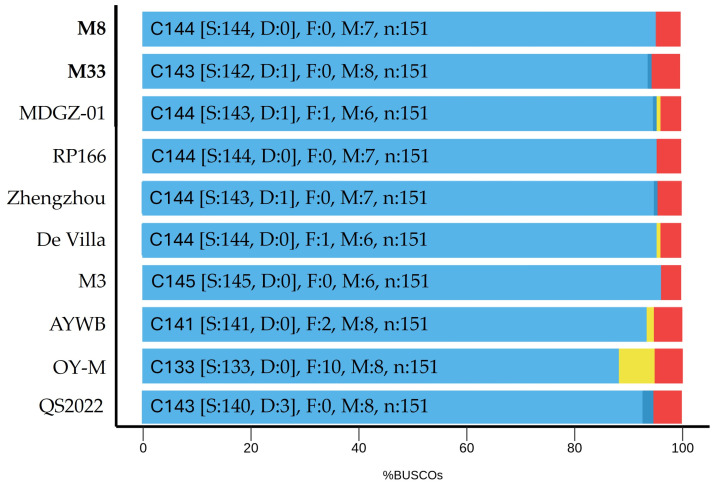
BUSCO results for the ‘*Ca*. P. asteris’ strains. Results are separated into (C) complete single copy orthologues comprising (S) single and (D) duplicated copy orthologues (blue), (F) fragmented orthologues (yellow), and (M) missing orthologues (red).

The final quality assessment of the hybrid system using BUSCO analysis revealed a total of 151 identified orthologues in each of the analysed genome sequences that match orthologues in the BUSCO database (Figure 2). This indicates high quality according to completeness concerning the evaluation of conserved orthologs within Mollicutes and supports membership of the reconstructed chromosomes in the AY group.

### 3.3. Genomic Benchmarks of the Taxon ‘Ca. P. asteris’

**Figure 3:**
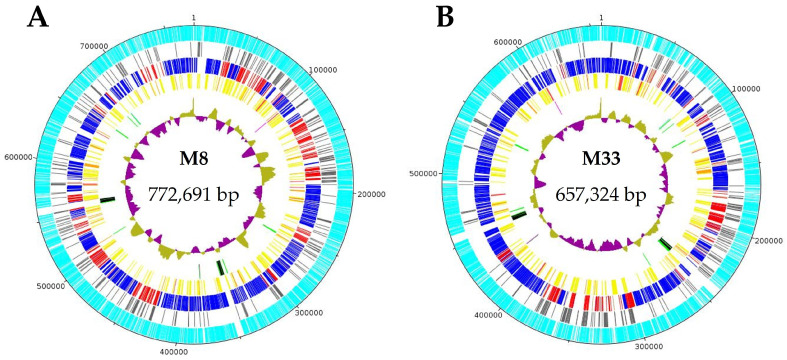
Genomic organisation of the circular chromosomes of ‘*Ca*. P. asteris’ strains M8 (**A**) and M33 (**B**). Circular patterns (from outside): 1 (outer ring), scale in base pairs of the chromosome; 2 (cyan), predicted protein-coding sequences; 2 (grey), hypothetical proteins; 3 (multi-coloured), predicted orthologues shared with all investigated genomes of the asteris group (dark blue), unique protein-coding sequences(brown), predicted paralogs (red); 4 (multi-coloured), predicted membrane proteins: with only a signal peptide (orange), only transmembrane domains (yellow), and both signal peptide and transmembrane domains (mid red); 5 (multi-coloured), RNAs: Signal recognition particle RNA and RNase P RNA component class B (magenta), transfer RNAs (green), and rRNA operons (black),transfer-messenger RNAs (light blue); 6 G+C skew (olive and pink).

The circular chromosomes of M8 and M33 differ by ~115 kb bp in size (Table 2, Figure 3). M8, with a chromosome size of ~773 kb, is among the larger chromosomes of the AY group (an average of 734.4 kb). For instance, ‘*Ca*. P. asteris’ Zhengzhou possesses the longest chromosome sequence, measuring roughly 892 kb, while M33, with a chromosome size of 657,324 bp, represents strains with smaller chromosome types. Among these, M3 has the smallest chromosome, with a length of approximately 576 kb. The total number, i.e., 741 CDS, in the M8 genome also differed compared to ‘*Ca*. P. asteris’ strain M33, which had 595 CDS (Table 2, Figure 3). However, the coding density of 0.958 kb/gene for strain M8 is above average (0.909 kb/gene). Moreover, strain M33 showed the lowest G+C content among the AY group, with a value of 26.8%, in contrast to other AY group members whose G+C content ranged from 27% to 29% (an average of 27.79%). Structural RNAs are encoded including the typical two rRNA operons and 32 tRNAs of phytoplasmas, but also *rnpB*, *ffs,* and *ssrA* [52]. M8 and M33, harbour a plasmid, a feature that is not described for all complete genomes within the AY group. In addition to strains M8 and M33, single plasmids were identified in strains OY-M, De Villa, and Zhengzhou, whereas AYWB had four reported plasmids. The QS2020 strain exhibited the highest coding density within the AY group, with 0.981 CDS/kb (Table 2). In summary, the completely reconstructed genomes of M8 and M33 display typical genomic features associated with the AY group.

### 3.4. Phylogenetic Analysis

#### 3.4.1. Average Nucleotide Identity

**Figure 4:**
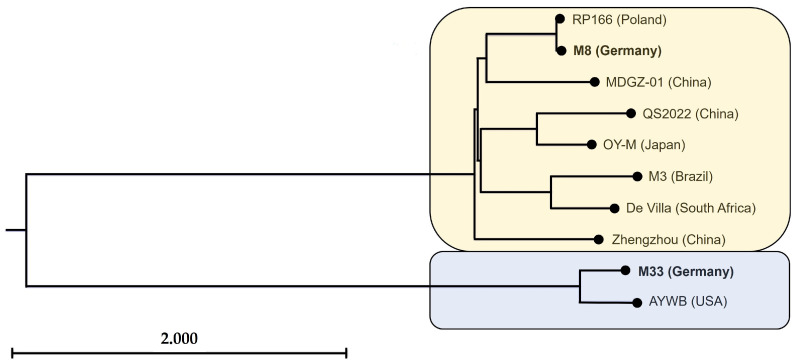
Phylogenetic tree based on ANI comparison from the analysed chromosomes of ‘*Ca*. P. asteris’ strains using the neighbour-joining method. Branch lengths are measured via the number of substitutions per digit.

The chromosomes of strains M8, M33, and the other analysed complete chromosomes of the AY group underwent ANI analysis, resulting in the formation of two distinct clusters. Despite originating from the same field, M8 and M33 clustered into different groups and this analysis did not reveal any obvious correlation in the geographical distribution of ‘*Ca*. P. asteris’ strains (Figure 4).

The strains M33 and AYWB displayed identity values ranging from 92% to 94% when compared to the other chromosomes within the AY group (Table 3), which is less than the 95% threshold recommended for taxon affiliation in recent requirements for the taxonomic revision of the genus ‘*Ca*. Phytoplasma’ [47].

**Figure 5:**
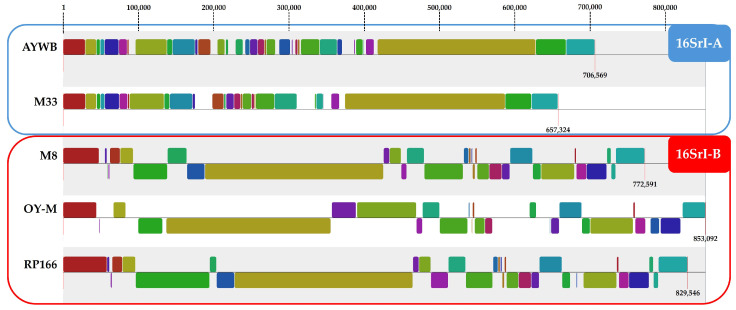
Sequence synteny analysis of the ‘*Ca*. P. asteris’ strains using Mauve. The outer blue and red box contain selected representatives of the respective ANI clusters of ‘*Ca*. P. asteris’ M8 and M33 as well as their associated 16SrI subgroups. Inner blocks with identical colours show sequence synteny.

Analyses highlight the assignment of these two strains to different clusters which may represent separate taxons, which is in accordance with the results of former studies using ANI analysis to investigate genomic divergence among 16SrI phytoplasmas [53]. The division ‘*Ca*. P. asteris’ strains into two groups via ANI analysis is further supported by blocks of conserved sequence synteny observed in Mauve (Figure 5).

#### 3.4.2. Single Gene Analysis Supports Cluster Formation

**Figure 6:**
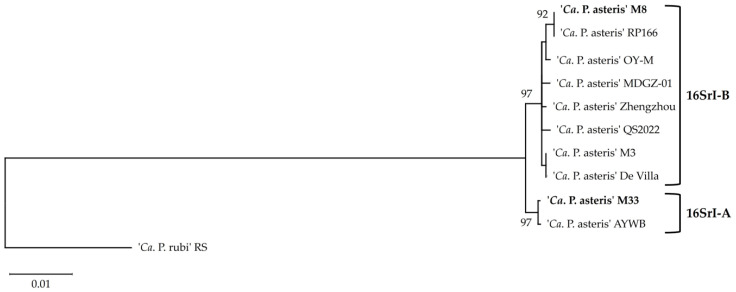
Phylogenetic tree of the ‘*Ca*. P. asteris’ strains constructed using the maximum likelihood method, using 16S rDNA sequences of the employing ‘*Ca*. P. rubi’ strain RS as the outgroup. Numbers on the branches are bootstrap values obtained for 1000 replicates (only values above 70% are shown). Strains from this study and their corresponding 16SrI subgroup are highlighted in bold.

ANI analysis revealed identities lower than 95% for the ribosomal cluster 16SrI-A comprising the strains M33 and AYWB compared to the other AY group members, which indicates a questionable affiliation of this cluster to the provisional taxon ‘*Ca*. P. asteris’. The phylogenetic split is also supported by the analysis of the 16S rRNA gene with both maximum likelihood (Figure 6) and neighbour-joining method (Figure S1). The 16S rRNA phylogeny assigned the M33 and M8 strains to I-A and I-B ribosomal subgroups, respectively [53].

The AY phytoplasma divergence was presented by several previously performed sequence analyses [53,54,55]. To verify if these results are also reflected at the single-gene level; a sequence identity was calculated based on the nucleotide sequences of the marker genes 16S rRNA, *tufB*, *groEL*, *secA*, and *secY*, and a chromosomal region containing the genes *rplV* and *rpsC* (Table 4). The pairwise values of the sequence identity matrix for the 16S rRNA gene showed no significant deviation from the threshold value of 98.65% of phytoplasmas [47] for the 16SrI-A group containing the strains M33 and AYWB. Pairwise comparison of the chromosomal region spanning from the gene *rplV* to *rpsC* resulted only in the AY strains DeVilla, M3, and Zhengzhou, with identity values that crossed the threshold of 97.50% and did not support the ANI assignment. Sequence identity analysis with *secY* appeared for strain M33 and AYWB identity values crossed the threshold of 95% and therefore supported the ANI cluster assignment. Similar support was achieved with the analysis of the genes *secA*, *groEL,* and *tufB*. Even though the threshold identity values were higher than the suggested thresholds, the values exceeded the threshold values, albeit to a minimal extent only. To confirm a significant overlap in species membership ‘*Ca*. P. rubi’ strain RS [56] was used as an outgroup in the phylogenetic analysis and exhibited significant divergence from the AY group clusters for all elicited markers compared to the cluster formed by strains M33 and AYWB (Table 4).

### 3.5. Pan-Genome Analyses

A comparison of the shared and unique features of the AY group was conducted via pan-genome analysis. In total 6717 CDS were used as queries for the ortholog prediction. Out of this total, 6505 CDS (96.8%) were assigned to 726 orthogroups with at least two members. The pairwise comparison of shared orthologs for each strain illustrates that ‘*Ca*. P. asteris’ M33 and AYWB shared the highest number of orthogroups (Table 5), whereas the carrot strains shared 401 orthogroups comprising a set of 503 proteins for M33 and 535 for M8, representing 84.5% and 72.2%, respectively. However, 326 single-copy orthologs were predicted to be shared by the asteris strains.

Therefore, the pan-genome analysis illustrated again the impact of paralog-associated information and supported the phylogenetic cluster assignment obtained from ANI, whole chromosome synteny, and single-gene analysis, and supports previous analysis [53]. It is notable that despite the number of shared orthologs the number of strain-specific features is low, with 212 CDS for the ten asteris chromosomes in total.

The strains M33 and M8 encode for 47 and 41 CDS not encoded by the other strains (Table S1, Figure S2). The deduced hypothetical proteins are characterised by a transmembrane domain (M33: 21/47; M8, 10/41). Six and five hypothetical proteins carry a signal peptide alone and represent putative secreted proteins (M33: M33023_01430, M33023_01470, M33023_03890, M33023_04050, M33023_04100, M33023_0677; M8: QN326_01250, QN326_04910, QN326_06420, QN326_06560, QN326_07200). According to these findings, the pan-genome analysis indicated that phytoplasmas of the AY group mainly differ in terms of features associated with pathogen–host interactions.

Multi-copy genes were predicted to investigate whether the genome size of phytoplasmas is associated with the occurrence of multi-copy genes (Table 6). The chromosome of strain M3 represents the smallest chromosome of the investigated AY group members with ~576 kb encoding 36 multicopy genes, whereas the Zhengzhou strain, with the largest chromosome of ~892 kb, possesses 385. These gene numbers represent 3.37% and 24.6% of the chromosome length, highlighting the impact. Strain M8 codes for 188 and M33 for 112 multi-copy genes, which puts them in the midfield with 14.1% and 9% of their chromosome length, respectively.

The impressive number of multi-copy genes is also characterised by a decreased G+C content for many asteris chromosomes, with the exception of strain MDGZ-01. It has the highest G+C content of the chromosomes examined and is separated from the other multi-copy genes in the other asteris chromosomes by an increased G+C content, which suggests horizontal gene transfer event(s) (Table 6). If considering the encoded function of the multi-copy genes, it was found that they are predominantly associated with the mobilome; for instance, genes known to be present on transposable elements are also described as potential mobile units (PMUs) or with genes originating from phage insertions as previously described for asteris [17] and phytoplasma genomes [57,58]. Instability is also supported by the detection of replicative forms [59] and recently for the origin and the coding of phyllogen genes in phytoplasmas [60].

### 3.6. Functional Reconstruction and Comparison

#### 3.6.1. Key Metabolism and Membrane Transport

**Figure 7:**
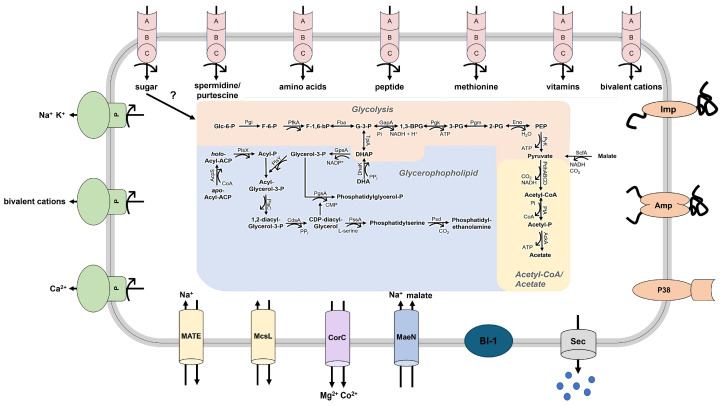
Schematic overview of the complete metabolic pathways suggesting membrane transport, and membrane proteins involved in pathogen–host interaction with ‘*Ca*. P. asteris’. Curved arrows indicate ATP hydrolysis. The unclear mechanism of phosphorylation and substrate supply for glycolysis have not been clarified in detail and have been labelled with a question mark in consequence.

The analysed chromosomes encode the core metabolic pathways of phytoplasmas, i.e., the core module of the glycolysis (starting from glucose 6-phosphate), pyruvate oxidation to acetyl coenzyme A (CoA) and acetate, and phosphatidylethanolamine biosynthesis as part of glycerophospholipid metabolism (Figure 7). In addition, both genomes encode the malate-acetate pathway conserved in phytoplasmas [57]. However, the asteris strains lack the utilisation of lactate as reported for 16SrV phytoplasmas [61]. In the chromosomes of M8 and M33, 53 and 39 CDS were identified that code for subunits for ABC transporters for sugars, amino acids, peptides, polyamides, vitamins, and bivalent cations. In total, seven putative ABC transporters were identified in the examined genomes. The ABC-type multiple sugar transport system is the only sugar uptake system identified in phytoplasmas, while a phosphoenolpyruvate-dependent sugar phosphotransferase system is missing and leaves the problem of substrate phosphorylation unsolved [57]. Furthermore, three P-type ATPases were encoded. All P-type ATPases were assigned to the function of exporting cations such as sodium, potassium, and calcium, or other non-selective bivalent cations. Symporters involved in regulation, like the magnesium-cobalt exporter CorC and the malate-sodium symporter MaeN, were identified. Conserved genes coding for antiporters were represented by the MATE family efflux transporter and the large conductance mechanosensitive channel MscL.

#### 3.6.2. Secretome and Characteristic Effector Proteins

M8 and M33 share with the other asteris strains the Sec-dependent secretion pathway encoded by the genes *secA*, *secE*, *secY*, *yidC*, *ffh*, and *ftsY* and representing the major functional secretion system identified within the investigated chromosomes of the AY group. Furthermore, components of the signal recognition particle (SRP) pathway were also identified. The SRP is involved in targeting ribosomes while translating and guiding them to the SecYEG pore complex of the Sec-dependent secretion pathway and targeting integral membrane proteins for co-translational integration into the membrane. The major SRP complex is formed by the SRP protein, a 4.5S RNA encoded by the gene *ffs* and the protein encoded by the gene *ffh* [62]. For a functional translocation system, the proteins YidC and FtsY are also needed, which were encoded in all AY genomes [63]. In total, 252 proteins for M8 and 231 proteins for M33 were predicted to be secreted by or integrated into the membrane. Predictions of the potential secretome with Phobius [43] comprised 43 proteins for the M8 strain and 33 for M33 (Table 7), which contained only a signal peptide domain (SP), 51 of which (67.11%) were shared by M8 and M33. Within the M8 chromosome, 201 proteins contained only transmembrane (TM) domain(s), whereas, for M33, 191 proteins were found, 297 (75.77%) of which were shared by strains M8 and M33. Eight proteins that had both (SP+TM) were predicted in M8 and seven were predicted in M33 (Figure 3). M8 and M33 shared eight out of a total of 15 proteins containing the SP and TM domains. Protein sequences that possess a signal peptide only were further investigated to identify the hitherto well-described and experimentally approved effector proteins TENGU, SAP05, SAP11, and SAP54. For all analysed chromosomes of the AY group, a TENGU gene coding for the effector was found, which indicates that *tengu* is conserved encoded in the AY group, which is in accordance with previous findings [53]. However, SAP05, SAP11, and SAP54 were not consistently encoded by the analysed complete ‘*Ca*. P. asteris’ chromosomes (Table S2). Notably, the genes coding for the effectors SAP11 and SAP54 were not identified for strain M33 whereas M8 encodes both genes. The lack of SAP11 stands in accordance with earlier findings from field studies supporting their absence in the 16SrI-B group [64]. Similar to the multi-copy gene analysis, these results also indicate that the numbers of genes coding for secreted or putative secreted proteins are higher within the larger chromosomes than in the smaller ones. This also supports the claim that interaction with the mobilome influences chromosome size, because secreted proteins such as the effectors from the SAP group are often present on transposable elements [59].

#### 3.6.3. Immunodominant Membrane Proteins

The immunodominant membrane protein (Imp) and the antigenic membrane protein (Amp) are involved in physical interactions with the respective host organism of phytoplasmas. Imp is suggested to interact with the host plant actin filament, whereas Amp interacts with the actin filaments and the beta subunit of the ATPase within the insect environment [65,66,67,68]. Our analysis showed that the genes *amp* and *imp* were encoded in all AY group members. Konnerth and colleagues showed the genomic context for *imp*, which is directly adjacent to the gene *pyrG* coding for CTP synthase and the *dnaG* that codes for a protein involved in DNA replication [68]. However, in this work, we identified a different genomic context for imp within the ANI cluster of ‘*Ca*. P. asteris’ AYWB and M33, whereby only *dnaG* was adjacent. In contrast to the other ANI cluster comprising ‘*Ca*. P. asteris’ M8, OY-M, M3, RP166, De Villa, MDGZ-01, and Zhengzhou, this cluster has a pseudogene assigned to glucose-1-phosphatase flanked to *imp*. For *amp*, the same genomic context was found as reported by Konnerth and associates [68]. This region possesses the bordering genes *nadE* and *groEL*, as well as *amp*.

#### 3.6.4. Adhesine P38

Another important group of cell surface factors comprises adhesins, which have hitherto been poorly studied in phytoplasmas. The adhesine P38 is suggested to interact with the insect host, as well as weakly with the plant host. Adhesin P38 was first described within the genome of ‘*Ca*. P. asteris’ OY-M [69]. A gene encoding adhesine P38 was found in all chromosomes of the analysed strains and showed a conserved genomic context. Flanking genes *pyk* and *pepV* coded for pyruvate kinase and the dipeptidase PepV.

#### 3.6.5. Bax-Inhibitor 1

Bax-inhibitor 1 (BI-1) is a protein that has been characterised to reduce programmed cell death (PCD) [70] and it has also been suggested as being present in phytoplasma genomes [71]. However, how this PCD suppressor works in phytoplasmas is still not clear. Bax-inhibitor 1 was found in all analysed AY genomes. Within the chromosome of strain M33, two identical copies of the BI-1 gene were encoded, whereas the other strains showed only single genes. All genes coding for BI-1 revealed a conserved genomic context and were flanked by the genes *tufB*, coding for the elongation factor Tu, and *rsmG* encoding a 16S rRNA (guanine(527)-N(7))-methyltransferase. Annotations for *rsmG* of ‘*Ca*. P. asteris’ OY-M, AYWB, and M3 are biased compared to other AY group members and are deposited as *gidB*.

#### 3.6.6. Superoxide Dismutase

Another notable feature is a gene-encoding superoxide dismutase (SOD), an important enzyme thought to be involved in protecting phytoplasmas against the plant defence response in the form of an oxidative burst created by enhanced reactive oxygen species (ROS) production [72,73]. Within the analysed AY genomes, a manganese/iron-dependent SOD was identified with a conserved genomic context, flanked by the genes *pdhA* coding for the pyruvate dehydrogenase E1 alpha subunit and *nusB*, which encodes a protein involved in transcription termination and antitermination.

### 3.7. Extrachromosomal Elements

**Figure 8:**
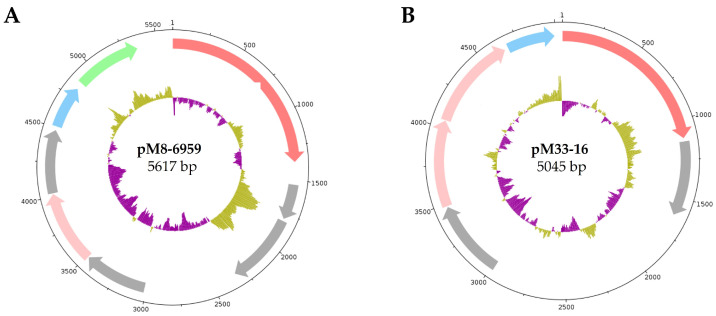
Plasmid map of pM8-6959 (**A**) and pM33-16 (**B**). Colour code: *rep* coding for the replication protein (red); *ssb* coding for single-strand binding protein (blue); putative secreted proteins (green); genes coding for putative copy number controlling proteins (pink); (grey) CDS assigned as hypothetical proteins.

Two plasmids, named pM8-6959 and pM33-16, were identified (Figure 8). The plasmids showed similar sequence lengths, with 5617 bp for pM8-6959 and 5045 bp for pM33-16. Eight ORFs were predicted for pM8-6959, whereas for pM33-16 six ORFs were identified. The exact positions of the OriC were not identified. The encoded *rep* genes were set at position 1 of the plasmids. In addition to, the replication-associated genes *rep* and *ssb*, a gene for a putative transmembrane domain-containing protein (QN326_00080) were found on pM8-6959, whereas for pM33-16, no encoded secreted proteins were identified. Moreover, genes that were assigned as putative copy number control proteins were found on both plasmids (QN326_00050; M33023_00050; M33023_00060). All remaining ORFs were assigned to hypothetical proteins that are not characterised by their function. Both plasmids were compared with each other and with all AY genomes at nucleotide and amino acid levels to identify potential interactions between the genomes in the form of horizontal gene transfer. The analysis showed no significant match at either the nucleotide or amino acid level. Results from the comparisons of the plasmids pM8-6959 and pM33-16 with the plasmid sequences from the other analysed complete genomes of ‘*Ca*. P. asteris’ showed that the *rep* and *ssb* gene sequences of pM8-6959 had the highest similarities with genes encoded on the plasmids of the onion-yellows (OY) group, whereas the sequences of the genes *rep* and *ssb* of pM33-16 shared the highest consensus with the plasmids pAWYB-I to IV of ‘*Ca*. P. asteris’ AYWB. This supports the assignment of M33 to 16SrIA and M8 to the 16Sr-IB group also at the extrachromosomal level.

## 4. Discussion

We added two complete genomes to the provisional taxon ‘*Ca*. P. asteris’. The strains ‘*Ca*. P. asteris’ M33 and M8—originating from the same field and host variety—were assigned to different phylogenetic clusters. The clusters reflect the phytoplasma phylogeny with the clearly diverged 16SrI-A and I-B subclades [74]. In view of this, an examination of the taxon status was proposed [53]. ANI cluster formation was also supported by single-gene phylogeny and whole-chromosome synteny analysis. An ongoing debate on the taxonomic revision of the genus ‘*Candidatus* Phytoplasma’ has recently started to consider ANI identity values, which again underlines the importance of this method for the taxonomic classification of newly discovered phytoplasma species. Moreover, the ANI method has been suggested as shifting the gold standard for the classification of prokaryotic species, especially in the era of big data, with an extreme increase in complete prokaryotic genome sequences [47,75,76,77,78]. In contrast to the three strains MDGZ-01, which infect mulberry (*Morus alba*), Zhengzhou [21], which is associated with paulownia (*Paulownia fortunei*), and QS2022 [18], which has been reported in lettuce (*Lactuca sativa*) in China, the M8 and M33 strains originated from the same field and host plant. These results are consistent with the occurrence of different 16SrI subgroups on individual fields during the season. Furthermore, our research confirms the disparities in the coded effectors of the SAP group within the 16SrI groups, which were illustrated by Clements and colleagues [64]. The phylogenetic analysis did not provide information on a shared geographical origin regarding the introduction into Germany (Figure 4). M8 shows a close relationship with ‘*Ca*. P. asteris’ RP166, which causes rapeseed phyllody in Poland; this may hint at the geographical location. The genetic distance of M8 and M33 might be linked to different vector populations or species. Cicadas of the genus *Macrosteles* are potential vectors. Another study from Germany showed that *Macrosteles sexnotaus*, *M*. *laevis* and *M*. *cristatus* caught in carrot fields infected with ‘*Ca.* Phytoplasma asteris’ also carried the pathogen, indicating that these cicadas are possible vectors [23].

Phytoplasmas have a narrow host range as far as their insect hosts are concerned, and since transovarial transmission is not a common case [79], the presence of an insect host is a crucial factor in their survival in the natural environment [80,81,82,83,84,85,86,87,88]. It cannot be excluded that this is also determined by the plasmids. Ishii and colleagues, for instance, provided evidence that the plasmid pOYNIM of ‘*Ca*. P. asteris’ OY-NIM had lost its *orf3* and could therefore not be transmitted by its insect vector. The plasmid sequences of pM8-6959 and pM33-16 encoded no similar sequence to *orf3*. An exact functional characterisation of this ORF was not provided, but it was established that *orf3* encodes a transmembrane protein representative on the cell surface [81,82,83]. Our results indicate that only the plasmid pM8-6959 possesses a gene coding for a protein with a length of 160 amino acids, which, according to Phobius prediction, is a transmembrane protein (QN326_00080), thus indicating that this gene could be involved in pathogen–host interaction (Figure 8). However, the exact role of this protein is still not clear, and whether the two strains ‘*Ca*. P. asteris’ M8 and M33 share one insect vector remains unclear. It has also been reported that phytoplasma transmission is possible through seed material from infected plants, which has been shown for carrots, and other important crop plants such as corn, (*Zea mays*), tomatoes (*Solanum lycopersicum*), winter oilseed rape (*Brassica napus*), and limes (*Citrus aurantifolia*) [4,89,90,91]. Mixing of different seed lots in the field of origin of M8 and M33 cannot be excluded but is unlikely to be the reason for the occurrence of the two different subgroups in one field since seed transmission is a rare case. Seed exports may represent a crucial issue considering the geographical distribution of these phytoplasmas, due to the fact that phytoplasmas in propagating material are not considered a risk either in the quarantine protocols of plant protection or by seed producers [79]. It is notable that the sampling field for M8 and M33 had poplar trees in their surroundings. Poplar trees have been reported as natural host plants for phytoplasmas in Europe, including black poplar (*Populus nigra* ‘Italica’ and *P*. *canadensis*) in Bulgaria, the Netherlands, Croatia, and Serbia; grey poplar (*P*. *canescens*) and white poplar (*P*. *alba*) from France and trembling poplar (*P*. *tremula*) in Germany. For France, Germany, and Serbia, Populus witches’ broom (PopWB) disease has been assigned to phytoplasmas of the aster yellows group [24,92,93,94,95,96,97,98]. Moreover, this was also demonstrated for some weed plants such as wild carrot (*Daucus carota* subsp. *carota*), hemlock (*Conium maculatum*), coast tarweed (*Madia sativa*), field bindweed (*Convolvulus arvensis*), and field madder (*Sherardia arvensis*). Therefore, screening for ‘*Ca*. P. asteris’ M8 and M33 and potential vectors associated with the poplar trees and weed plants in the tested cropping area should be considered.

## 5. Conclusions

In this study, we added two complete genome sequences to the provisional taxon ‘*Ca*. P. asteris’ sharing the host carrot. We confirmed the phylogenetic differentiation of the 16Sr I-A and I-B subclades in this taxon at the whole-genome level. Moreover, it was confirmed that the basic repertoire of genes coding for proteins with metabolic functions is highly conserved. Genomic plasticity regarding chromosome size is therefore not associated with extended metabolic functions but rather with duplication events and mobilome interactions. Pan-genome analysis of the AY group established that the unique features contributed to phytoplasma effector variability.

## Figures and Tables

**Table 1:** Canu hybrid assembly statistics of the complete genomes of ‘*Ca*. P. asteris’ M8 and M33.

	M8	M33
Assembly input		
Selected/mapped Illumina reads (%)	223,496 (10.6)	480,718 (22.2)
Average read length (nt)	250	250
SMRT reads total	191,988	105,166
Average read length (nt)	8694	9275
Incorporated reads		
No. of Illumina & SMRT reads used for chromosome reconstruction (sequencing coverage)	10,116	11,776
	(115.86-fold)	(176.15-fold)
No. of reads used for plasmid reconstruction (sequencing coverage)	307	45
	(95.09-fold)	(11.97-fold)
Genome organization (complete)	One circular chromosome, one plasmid	One circular chromosome, one plasmid

**Table 2:** Genomic benchmarks of the analysed complete chromosomes within the taxon ‘*Ca*. P. asteris’ according to annotation.

16Sr-Group	Strain	Country of Origin	Plant Host	Length (bp)	G+C Content (%)	CDS (+ Pseudo Genes)	Coding Density (per kb)	Acc. no. ^1^
**16SrI-A**	**M33**	**Germany**	**Carrot**	**657,324**	**26.79**	**595 (51)**	**0.905**	**CP128397.1**
	AYWB	USA	Lettuce	706,569	26.89	586 (62)	0.829	NC_007716.1
**16SrI-B**	**M8**	**Germany**	**Carrot**	**772,691**	**27.87**	**741 (77)**	**0.958**	**CP128414.1**
	OY-M	Japan	Onion	853,092	27.76	752 (-)	0.881	AP006628.2
	M3	Brazil	Maize	576,118	28.46	485 (-)	0.841	CP015149.1
	DeVilla	South Africa	Periwinkle	600,116	28.44	545 (10)	0.908	CP035949.1
	RP166	Poland	Rapeseed	829,546	27.70	753 (-)	0.907	CP055264.1
	Zhengzhou	China	Paulownia	891,641	27.35	906 (68)	1.016	NZ_CP066882.1
	MDGZ-01	China	Mulberry	622,358	29.09	535 (10)	0.859	NZ_CP085837.1
	QS2022	China	Lettuce	834,303	27.57	819 (-)	0.981	CP120448.1

^1^ Data from RefSeq were used with regard to continuous curation (indicated by underline) if available. Data from this study is highlighted bold.

**Table 3:** ANI matrix of the compared ‘*Ca*. P. asteris’ chromosome sequences.

Strain	M33	AYWB	M8	OY-M	M3	DeVilla	RP166	Zhengzhou	MDGZ-01	QS2022
**M33**		99.44	**93.19**	**92.97**	**92.88**	**92.85**	**93.29**	**92.95**	**92.93**	**92.91**
AYWB	99.44		**93.20**	**93.00**	**92.80**	**92.80**	**93.19**	**92.91**	**92.93**	**92.81**
**M8**	**93.19**	**93.20**		99.02	98.54	98.64	99.93	98.66	98.91	98.44
OY-M	**92.97**	**93.00**	99.02		98.44	98.49	99.01	98.49	98.70	99.12
M3	**92.88**	**92.80**	98.54	98.44		99.15	98.56	98.24	98.37	98.23
DeVilla	**92.85**	**92.80**	98.64	98.49	99.15		98.63	98.29	98.40	98.31
RP166	**93.29**	**93.19**	99.93	99.01	98.56	98.63		98.64	98.91	98.45
Zhengzhou	**92.95**	**92.91**	98.66	98.49	98.24	98.29	98.64		98.35	98.12
MDGZ-01	**92.93**	**92.93**	98.91	98.70	98.37	98.40	98.91	98.35		98.51
QS2022	**92.91**	**92.81**	98.44	99.12	98.23	98.31	98.45	98.12	98.51	

Identity values in percentages. Bold values show identities crossing the species affiliation threshold [47].

**Table 4:** Sequence identity matrices for the genes 16S rRNA, *tufB*, *groEL*, *secA*, *secY,* and the genomic region spanning between *rplV* to *rpsC*.

16SrRNA	M33	AYWB	M8	OY-M	M3	DeVilla	RP166	Zhengzhou	MDGZ-01	QS2022	RS
**M33**	**-**	99.4	99.3	98.7	99.2	98.8	99	98.9	98.8	99.1	**89.1**
AYWB	99.4	-	98.8	99.2	98.6	99.2	98.5	99.4	99.4	**98.6**	**89.4**
**M8**	99.3	98.8	**-**	99.1	99.4	99	99.7	99.2	99.1	99.4	**88.9**
OY-M	98.7	99.2	99.1	-	98.8	99.6	98.8	99.6	99.6	98.8	**89.3**
M3	99.2	**98.6**	99.4	98.8	-	99	99.7	99	99	99.8	**88.7**
DeVilla	98.8	99.2	99	99.6	99	-	98.8	99.6	99.5	98.8	**89.5**
RP166	99	98.5	99.7	98.8	99.7	98.8	-	98.9	98.8	99.6	**88.6**
Zhengzhou	98.9	99.4	99.2	99.6	99	99.6	98.9	-	99.8	99	**89.3**
MDGZ-01	98.8	99.4	99.1	99.6	99	99.5	98.8	99.8	-	98.9	**89.2**
QS2022	99.1	**98.6**	99.4	98.8	99.8	98.8	99.6	99	98.9	-	**88.6**
RS	**89.1**	**89.4**	**88.9**	**89.3**	**88.7**	**89.5**	**88.6**	**89.3**	**89.2**	**88.6**	-
***tufB***	**M33**	AYWB	**M8**	OY-M	M3	DeVilla	RP166	Zhengzhou	MDGZ-01	QS2022	RS
**M33**	-	99.8	**96.7**	**96.7**	**96.7**	**96.7**	**96.7**	**96.4**	**96.7**	**96.8**	**68**
AYWB	99.8	-	**96.8**	**96.8**	**96.8**	**96.7**	**96.8**	**96.5**	**96.8**	**96.9**	**68**
**M8**	**96.7**	**96.8**	-	100	99.6	99.4	100	99.6	100	99.7	**67.8**
OY-M	**96.7**	**96.8**	100	-	99.6	99.4	100	99.6	100	99.7	**67.8**
M3	**96.7**	**96.8**	99.6	99.6	-	99.7	99.6	99.3	99.6	99.5	**67.8**
DeVilla	**96.7**	**96.7**	99.4	99.4	99.7	-	99.4	99	99.4	99.3	**67.9**
RP166	**96.7**	**96.8**	100	100	99.6	99.4	-	99.6	100	99.7	**67.8**
Zhengzhou	**96.4**	**96.5**	99.6	99.6	99.3	99	99.6	-	99.6	99.4	**67.7**
MDGZ-01	**96.7**	**96.8**	100	100	99.6	99.4	100	99.6	-	99.7	**67.8**
QS2022	**96.8**	**96.9**	99.7	99.7	99.5	99.3	99.7	99.4	99.7	-	**67.8**
RS	**68**	**68**	**67.8**	**67.8**	**67.8**	**67.9**	**67.8**	**67.7**	**67.8**	**67.8**	-
***groE*** *L*	**M33**	AYWB	**M8**	OY-M	M3	DeVilla	RP166	Zhengzhou	MDGZ-01	QS2022	RS
**M33**	-	99.8	**97**	**97.1**	**96.8**	**96.9**	**97**	**96.8**	**96.8**	**96.8**	**69.5**
AYWB	99.8	**-**	**97.2**	**97.2**	**97**	**97**	**97.2**	**96.9**	**96.9**	**97**	**69.5**
**M8**	**97**	**97.2**	-	99.9	99.8	99.7	100	99.7	99.7	99.6	69.4
OY-M	**97.1**	**97.2**	99.9	-	99.7	99.8	99.9	99.6	99.6	99.5	69.4
M3	**96.8**	**97**	99.8	99.7	-	99.8	99.8	99.5	99.5	99.4	69.5
DeVilla	**96.9**	**97**	99.7	99.8	99.8	-	99.7	99.5	99.5	99.3	69.4
RP166	**97**	**97.2**	100	99.9	99.8	99.7	-	99.7	99.7	99.6	69.4
Zhengzhou	**96.8**	**96.9**	99.7	99.6	99.5	99.5	99.7	-	99.5	99.3	69.3
MDGZ-01	**96.8**	**96.9**	99.7	99.6	99.5	99.5	99.7	99.5	-	99.3	69.6
QS2022	**96.8**	**97**	99.6	99.5	99.4	99.3	99.6	99.3	99.3	-	69.4
RS	**69.5**	**69.5**	**69.4**	**69.4**	**69.5**	**69.4**	**69.4**	**69.3**	**69.6**	**69.4**	-
***secA***	**M33**	AYWB	**M8**	OY-M	M3	DeVilla	RP166	Zhengzhou	MDGZ-01	QS2022	RS
**M33**	-	99.9	**95.2**	**95.2**	**95.1**	**95.1**	**95.2**	**95.1**	**95.3**	**95.2**	**67.7**
AYWB	99.9	**-**	**95.2**	**95.3**	**95.1**	**95.1**	**95.2**	**95.2**	**95.3**	**95.3**	**67.8**
**M8**	**95.2**	**95.2**	-	99.6	99.2	99.2	100	99.4	99.6	99.3	**67.7**
OY-M	**95.2**	**95.3**	99.6	-	99.3	99.3	99.6	99.5	99.6	99.4	**67.8**
M3	**95.1**	**95.1**	99.2	99.3	-	99.4	99.2	99.2	99.4	99.2	**68.1**
DeVilla	**95.1**	**95.1**	99.2	99.3	99.4	-	99.2	99.2	99.4	99.2	**67.9**
RP166	**95.2**	**95.2**	100	99.6	99.2	99.2	-	99.4	99.6	99.3	**67.7**
Zhengzhou	**95.1**	**95.2**	99.4	99.5	99.2	99.2	99.4	-	99.5	99.3	**67.8**
MDGZ-01	**95.3**	**95.3**	99.6	99.6	99.4	99.4	99.6	99.5	-	99.4	**67.8**
QS2022	**95.2**	**95.3**	99.3	99.4	99.2	99.2	99.3	99.3	99.4	-	**67.9**
RS	**67.7**	**67.8**	**67.7**	**67.8**	**68.1**	**67.9**	**67.7**	**67.8**	**67.8**	**67.9**	-
***secY***	**M33**	AYWB	**M8**	OY-M	M3	DeVilla	RP166	Zhengzhou	MDGZ-01	QS2022	RS
**M33**	-	99.8	**94.7**	**94.2**	**94.5**	**94.4**	**94.7**	**94.2**	**93.9**	**94.2**	**55**
AYWB	99.8	-	**94.9**	**94.4**	**94.6**	**94.6**	**94.9**	**94.4**	**94.1**	**94.3**	**55.2**
**M8**	**94.7**	**94.9**	-	99.4	99.3	99.3	100	99.1	99	99.3	**55.5**
OY-M	**94.2**	**94.4**	99.4	-	99.1	99.1	99.4	98.9	98.7	99.7	**55.4**
M3	**94.5**	**94.6**	99.3	99.1	-	99.5	99.3	98.8	98.7	99	**55.5**
DeVilla	**94.4**	**94.6**	99.3	99.1	99.5	-	99.3	99	98.7	99	**55.6**
RP166	**94.7**	**94.9**	100	99.4	99.3	99.3	-	99.1	99	99.3	**55.5**
Zhengzhou	**94.2**	**94.4**	99.1	98.9	98.8	99	99.1	-	98.5	98.8	**55.5**
MDGZ-01	**93.9**	**94.1**	99	98.7	98.7	98.7	99	98.5	-	98.7	**55.5**
QS2022	**94.2**	**94.3**	99.3	99.7	99	99	99.3	98.8	98.7	**-**	**55.3**
RS	**55**	**55.2**	**55.5**	**55.4**	**55.5**	**55.6**	**55.5**	**55.5**	**55.5**	**55.3**	-
***rplV-rpsC***	**M33**	AYWB	**M8**	OY-M	M3	DeVilla	RP166	Zhengzhou	MDGZ-01	QS2022	RS
**M33**	-	100	97.7	97.5	**97.1**	**97.3**	97.7	**97**	97.5	97.5	**63.6**
AYWB	100	-	97.7	97.5	**97.1**	**97.3**	97.7	**97**	97.5	97.5	**63.6**
**M8**	97.7	97.7	-	99.8	99.4	99.4	100	99.3	99.8	99.8	**63.2**
OY-M	97.5	97.5	99.8	-	99.2	99.2	99.8	99.2	99.6	99.6	**63.2**
M3	97.1	97.1	99.4	99.2	-	99.6	99.4	98.8	99.2	99.2	**63.1**
DeVilla	97.3	97.3	99.4	99.2	99.6	-	99.4	98.8	99.2	99.2	**63.2**
RP166	97.7	97.7	100	99.8	99.4	99.4	-	99.3	99.8	99.8	**63.2**
Zhengzhou	**97**	**97**	99.3	99.2	98.8	98.8	99.3	-	99.2	99.2	**63**
MDGZ-01	97.5	97.5	99.8	99.6	99.2	99.2	99.8	99.2	-	99.6	**63.1**
QS2022	97.5	97.5	99.8	99.6	99.2	99.2	99.8	99.2	99.6	-	**63.2**
RS	**63.6**	**63.6**	**63.2**	**63.2**	**63.1**	**63.2**	**63.2**	**63**	**63.1**	**63.2**	-

Identity values in percentages. Bold values show identities crossing the species affiliation threshold [47].

**Table 5:** Pairwise comparison of shared ‘*Ca*. P. asteris’ orthogroups.

Strain	M33	AYWB	M8	OY-M	M3	DeVilla	RP166	Zhengzhou	MDGZ-01	QS2022
**M33**	**-**	**421**	**401**	**382**	**370**	**370**	**398**	**381**	**365**	**403**
AYWB	421	-	410	389	377	373	411	388	376	404
**M8**	**401**	**410**	**-**	**437**	**418**	**432**	**597**	**448**	**420**	**470**
OY-M	382	389	437	-	399	411	437	441	407	498
M3	370	377	418	399	-	429	414	413	400	414
DeVilla	370	373	432	411	429	-	421	443	413	426
RP166	398	411	597	437	414	421	-	443	411	463
Zhengzhou	381	388	448	441	413	443	443	-	420	456
MDGZ-01	365	376	420	407	400	413	411	420	-	406
QS2022	403	404	470	498	414	426	463	456	406	-

Data from this study is highlighted in bold.

**Table 6:** Impact of multi-copy genes in complete asteris chromosomes.

16Sr-Group	Strain	All Genes	Multi-Copy Genes			
		No.	No.	% of All	Σ Length in bp	Chromosome (%)
**16SrI-A**	**M33**	**595**	**112**	**18.82**	**59,451**	**9.04**
	AYWB	586	155	26.45	85,755	12.14
**16SrI-B**	**M8**	**741**	**188**	**25.37**	**108,651**	**14.06**
	OY-M	752	251	33.38	185,037	21.69
	M3	485	36	7.42	19,425	3.37
	DeVilla	545	78	14.31	48,174	8.03
	RP166	753	269	35.72	177,861	21.44
	Zhengzhou	906	385	42.49	219,270	24.59
	MDGZ-01	535	66	12.34	46,800	7.52
	QS2022	819	291	35.53	172,767	20.71

Data from this study is highlighted in bold.

**Table 7:** Differentiation of proteins with respect to coding or absence of a signal and/or one or more membrane domain(s).

Strains	Cytosolic	Signal Peptide	Signal Peptide and Transmembrane Domaine	Transmembrane Domaine Only
**M33**	**369**	**33**	**7**	**186**
AYWB	399	31	9	147
**M8**	**498**	**43**	**8**	**192**
OY-M	517	37	12	186
M3	333	20	7	125
DeVilla	387	19	7	132
RP166	493	47	8	205
Zhengzhou	630	30	9	237
MDGZ-01	361	26	11	137
QS2022	519	44	11	245

Data from this study is highlighted in bold.

## Data Availability

Annotation for ‘*Ca*. P. asteris’ M8 and M33 were deposited in the GenBank database under accession numbers CP128397.1 and CP128414.1.

## Supplementary Materials

The following supporting information can be downloaded at: https://www.mdpi.com/article/10.3390/microorganisms12051016/s1. Table S1: CDS only encoded in ‘*Ca*. P. asteris’ strain M8 and M33, respectively, Table S2: Coding of the experimentally approved effector proteins within the asteris group, Figure S1. Phylogenetic tree of the ‘*Ca*. P. asteris’ strains were constructed using the neighbour-joining method, using 16S rDNA sequences of the employing ‘*Ca*. P. rubi’ strain RS as the outgroup. Numbers on the branches are bootstrap values obtained for 1000 replicates (only values above 70% are shown), Figure S2. Number of shared and unique CDS within the asteris group.
