# Supplementary material for: Divergence within the Taxon ‘Candidatus Phytoplasma asteris’ Confirmed by Comparative Genome Analysis of Carrot Strains
Source: Microorganisms. 2024 May 17;12(5):1016. doi: 10.3390/microorganisms12051016 (PMC11123874; doi:10.3390/microorganisms12051016)
Supplement: Supplementary file 1 [file microorganisms-12-01016-s001.zip › microorganisms-2948178-supplementary.pdf]

Table S1. Unique CDS of '*Ca*. P. asteris' strains M8 and M33.

Locus tag	Product
**M8**	
QN326_00710	hypothetical protein
QN326_01000	hypothetical protein
QN326_01250	putative secreted protein
QN326_02490	hypothetical protein
QN326_02740	hypothetical protein
QN326_03060	hypothetical protein
QN326_03110	hypothetical protein
QN326_03170	hypothetical protein
QN326_03370	hypothetical protein
QN326_03390	hypothetical protein
QN326_03550	hypothetical protein
QN326_04070	hypothetical protein
QN326_04150	hypothetical protein
QN326_04390	hypothetical protein
QN326_04480	hypothetical protein
QN326_04570	hypothetical protein
QN326_04590	hypothetical protein
QN326_04690	hypothetical protein
QN326_04910	putative secreted protein
QN326_04990	hypothetical protein
QN326_05220	hypothetical protein
QN326_05250	hypothetical protein
QN326_05410	hypothetical protein
QN326_05790	hypothetical protein
QN326_05890	hypothetical protein
QN326_06420	putative secreted protein
QN326_06560	putative secreted protein
QN326_06680	hypothetical protein
QN326_06710	hypothetical protein
QN326_06750	hypothetical protein
QN326_07180	hypothetical protein
QN326_07200	putative secreted protein
QN326_07280	hypothetical protein
QN326_07300	hypothetical protein
QN326_07370	hypothetical protein
QN326_07400	hypothetical protein
QN326_07450	hypothetical protein
QN326_07490	hypothetical protein
QN326_07710	hypothetical protein
QN326_08240	DEAD/DEAH box helicase
QN326_08580	hypothetical protein
**M33**	
M33023_00300	hypothetical protein
M33023_00330	hypothetical protein
M33023_00340	hypothetical protein
M33023_00350	hypothetical protein
M33023_00360	hypothetical protein
M33023_00420	hypothetical protein
M33023_00520	hypothetical protein
M33023_00910	hypothetical protein
M33023_01030	hypothetical protein
M33023_01150	hypothetical protein
M33023_01230	hypothetical protein
M33023_01390	hypothetical protein
M33023_01410	single-strand DNA-binding protein
M33023_01430	putative secreted protein
M33023_01470	putative secreted protein
M33023_01680	hypothetical protein
M33023_01690	hypothetical protein
M33023_02070	hypothetical protein
M33023_02300	hypothetical protein

M33023_02330	hypothetical protein
M33023_02350	hypothetical protein
M33023_02360	hypothetical protein
M33023_02370	hypothetical protein
M33023_02450	hypothetical protein
M33023_02470	hypothetical protein
M33023_02810	hypothetical protein
M33023_02840	hypothetical protein
M33023_03330	hypothetical protein
M33023_03520	hypothetical protein
M33023_03840	hypothetical protein
M33023_03890	putative secreted protein
M33023_04010	hypothetical protein
M33023_04050	putative secreted protein
M33023_04100	putative secreted protein
M33023_04840	hypothetical protein
M33023_04960	hypothetical protein
M33023_04980	hypothetical protein
M33023_05660	hypothetical protein
M33023_05800	hypothetical protein
M33023_05810	hypothetical protein
M33023_06010	hypothetical protein
M33023_06270	hypothetical protein
M33023_06460	hypothetical protein
M33023_06520	single stranded DNA binding protein
M33023_06600	hypothetical protein
M33023_06770	putative secreted protein
M33023_06780	hypothetical protein

*Unique proteins represent the CDS that were not assigned by Orthofinder.

Table S2. Coding of experimentally validated effector proteins within the asteris group.

**Strain**	**M33**	AYWB	**M8**	OY-M	M3	DeVilla	RP166	Zhengzhou	MDGZ-01	QS2022
**Locus tag**	M33023_	AYWB_RS	QN326_	PAM_	MBSPM3_v1c	EXT02_	RP166_	HGD80_RS	LJP71_RS	QS2022_
**Tengu**	02140	00785	06170	765	2160	01135	6200	01210	02000	4580
**SAP05**	00370	00160	00280	518			0280	03510		
**SAP11**		01770	00690	519	1080		0710			
**SAP54**		01055	00730	049			0750			0730

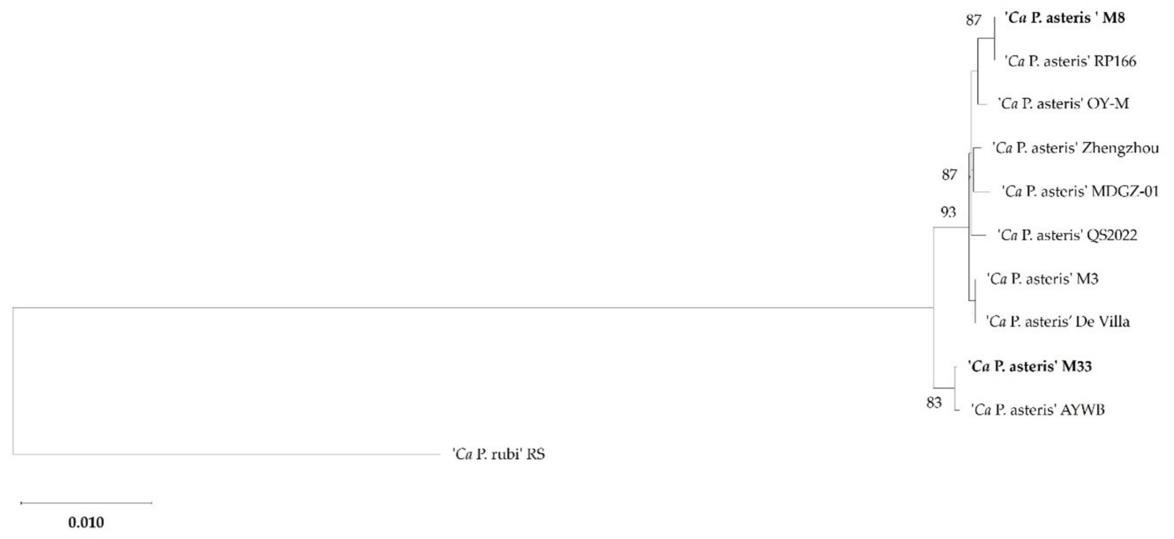

Figure S1. Phylogenetic tree of the '*Ca*. P. asteris' strains constructed using the neighbour-joining method, using 16S rDNA sequences of the employing '*Ca*. P rubi' strain RS as the outgroup. Numbers on the branches are bootstrap values obtained for 1000 replicates (only values above 70 % are shown).

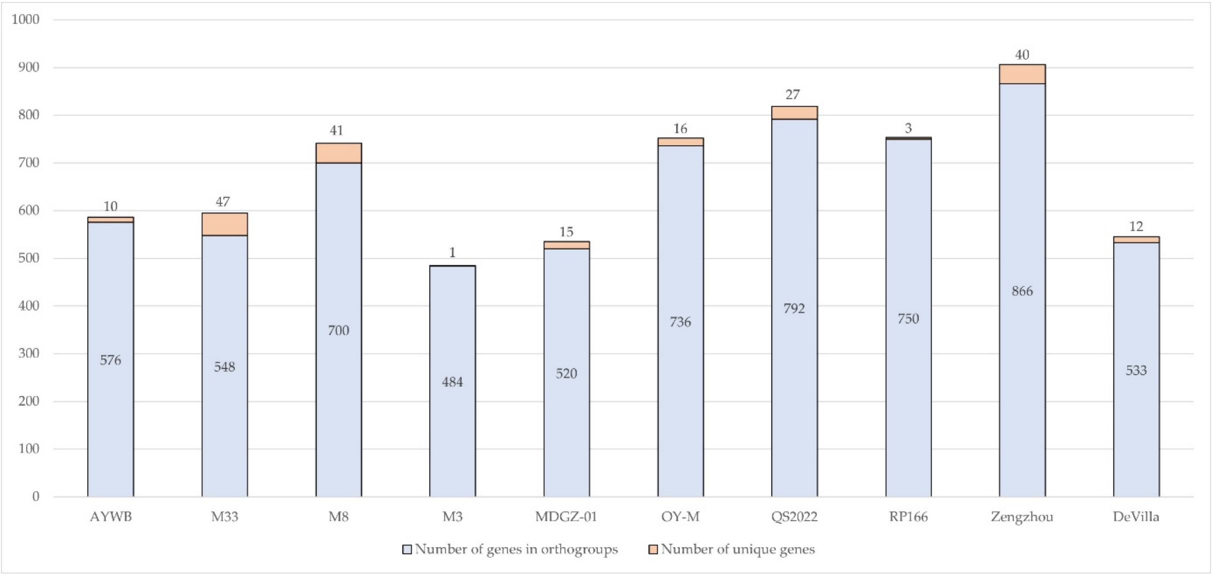

Figure S2. Number of shared and unique CDS within the asteris group.
